# Navigating Challenges in Biomedical Waste Management in India: A Narrative Review

**DOI:** 10.7759/cureus.55409

**Published:** 2024-03-02

**Authors:** Komal S Dhole, Sweta Bahadure, Gulshan R Bandre, Obaid Noman

**Affiliations:** 1 Pathology, School of Allied Health Sciences, Datta Meghe Institute of Higher Education and Research, Wardha, IND; 2 Pathology, Datta Meghe Medical College, Datta Meghe Institute of Higher Education and Research, Wardha, IND; 3 Microbiology, Jawaharlal Nehru Medical College, Datta Meghe Institute of Higher Education and Research, Wardha, IND

**Keywords:** waste handling, waste segregation, healthcare facility, impact india, biomedical waste management (bmwm)

## Abstract

Biomedical waste management (BMWM) in India poses significant challenges that demand thorough examination and strategic interventions. As the country's healthcare sector expands rapidly, proper management of biomedical waste becomes increasingly critical to safeguarding public health and environmental integrity. Biomedical waste, encompassing industrial waste, hospital waste, and waste from other healthcare facilities, poses a heightened risk of infection and injury compared to any other form of waste. A lack of understanding regarding safe medical waste disposal practices can be hazardous to one's health as well as the environment. To improve waste management practices in the country, we can suggest effective strategies and recommendations by developing a deeper understanding of the current situation. To manage medical waste effectively, healthcare professionals must be knowledgeable about and have experience with this process. This evaluation study provides a comprehensive overview of current BMWM methods in India, shedding light on the benefits, drawbacks, challenges, and areas for improvement in the healthcare waste management system. Several important facets of BMWM were highlighted by the literature research, including waste segregation, treatment techniques, and disposal options, as well as compliance and regulatory frameworks.

## Introduction and background

In global healthcare frameworks, effective management of waste from medical establishments, educational institutions, and laboratories is paramount. Proper biomedical waste management (BMWM) is essential to preventing environmental contamination and ensuring safety for both the public and medical personnel [1]. India faces significant challenges in managing biomedical waste due to its rapid development and expanding healthcare sector [2]. The production of biomedical waste has increased significantly across the country due to the exponential growth of medical facilities, the growing population, and improvements in healthcare technology [3]. As a result, improper waste management techniques are now a source of concern because they pose significant risks to both the environment and human health [4]. Environmental and healthcare experts have directed their attention toward advocating for the establishment of comprehensive and enduring BMWM frameworks within India [5]. In order to reduce the potential risk of infectious diseases, management of hazardous and non-hazardous biological waste is necessary [6]. The regulatory framework governing BMWM in India assesses its effectiveness in ensuring compliance and accountability among healthcare facilities and explores the adoption of advanced technologies and innovative practices in waste treatment and disposal to reduce the environmental impact of biomedical waste [7,8]. Understanding the advantages and disadvantages of current waste management techniques will lay the foundations for recommending evidence-based interventions and changes in policies that adhere to global best practices [9]. 

To address the urgent problem of biomedical waste in India, stakeholders must cooperate and act together [10]. By fostering a culture characterized by accountability and sustainability, the capacity to enhance the safety and well-being of the environment for both current and forthcoming generations is possible [11]. The purpose of this study is to assess and analyze India's current BMWM methods. This study assesses the benefits, drawbacks, challenges, and areas for developing the healthcare waste management system. The outcomes of this study are anticipated to provide healthcare leaders, waste management authorities, and other stakeholders with enhanced insights into the prevailing status of BMWM within India.

## Review

Method

A comprehensive review of the literature was conducted utilizing reputable databases, including PubMed, Google Scholar, Scopus, Web of Science, and Embase. The search encompassed articles released between 2005 and 2023, employing a specific set of search terms such as ("Biomedical waste") OR ("biomedical waste") AND ("Institution learning") OR ("institution learning") AND ("Institution teaching") OR ("biomedical teaching") OR ("Biomedical segregation") OR ("Biomedical handling") OR ("Biomedical technology-enhanced learning"). The final selection process adhered to a defined set of inclusion criteria: (1) origination as research articles, (2) peer-reviewed status, (3) availability of the complete text, (4) pertinence to the subject of biomedical waste, and (5) alignment with the specified timeframe for publication.

Article Screening

**Figure 1:**
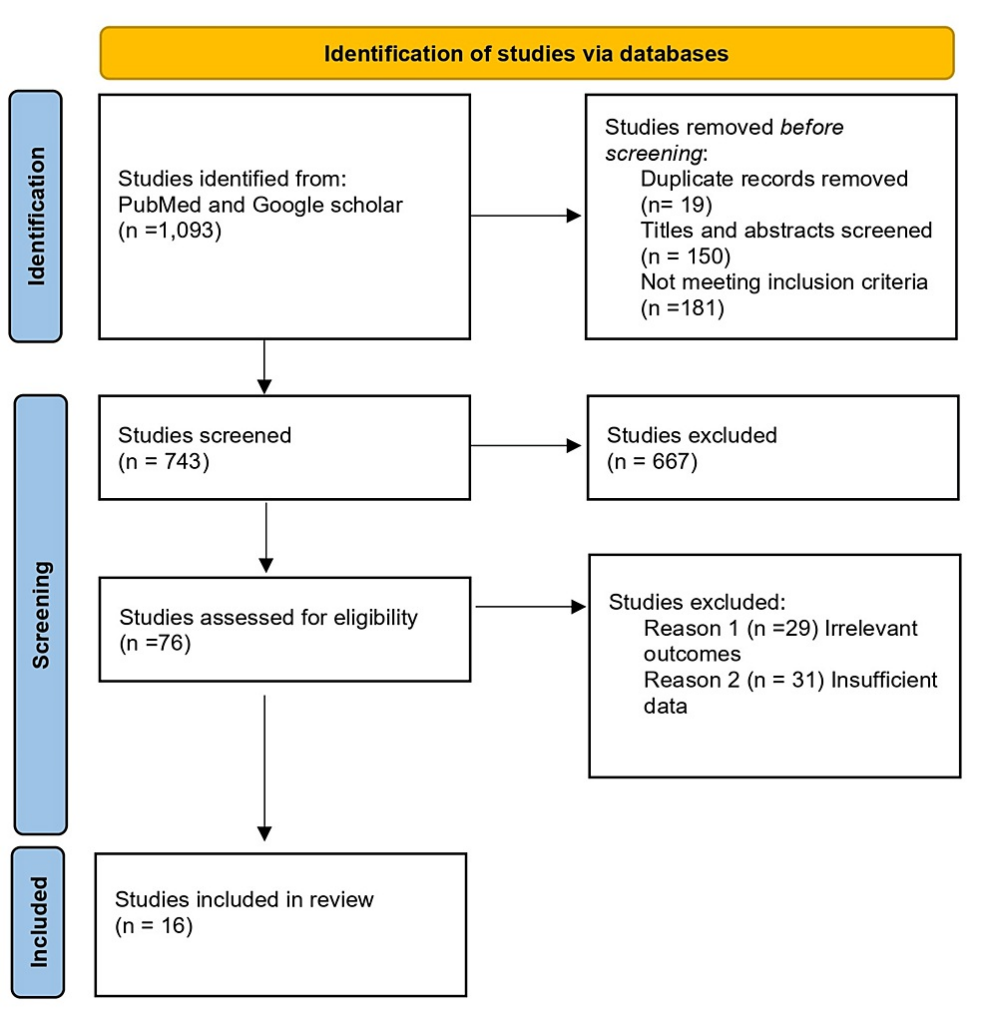
PRISMA flow diagram PRISMA: Preferred Reporting Items for Systematic Reviews and Meta-Analyses; n: number of studies.

After conducting the initial search, we identified 1093 articles in the searched databases. We then excluded duplicates (n=19) and performed an initial screening of titles and abstracts, which excluded another 667 articles. After the full-text screening of the remaining 76 articles, we excluded 60 articles for not meeting the inclusion criteria; either they were unrelated or some were for patient care, leaving 16 articles for the final review (Figure 1) [12].

Table 1 compiles various research investigations conducted on BMWM in healthcare environments across different regions of India. The focus of these inquiries is primarily on exploring the understanding, perspectives, and operational behaviors of healthcare professionals and auxiliary staff members in effectively managing biomedical waste. The studies identify gaps and areas for improvement in waste management practices. 

**Table 1:** Studies included in the study BMWM: biomedical waste management; PRHPs: private rural health providers; HCWM: healthcare waste management; HCWs: healthcare workers; BMW: biomedical waste.

S. No.	Reference	Year of publication	Study population	Study area	Outcome measure	Result
1	Pandit et al. [11]	2005	Doctors and auxiliary staff of hospitals	Gujarat	Biomedical waste management awareness and practices	Doctors have awareness about biomedical waste but lack details. Auxiliary staff have poor knowledge about biomedical waste.
2	Yadavannavar et al. [13]	2010	Teaching and non-teaching staff of hospitals	Bijapur	Knowledge, attitude, and practices regarding BMW management	334 employees were polled; 154 were educating staff, and 180 were not. Similar distinctions in attitudes and behaviors on BMW management were found between teachers and non-teaching staff (P<0.01).
3	Mathur et al. [14]	2011	Hospital staff (bed capacity >100) of Allahabad city	India	Knowledge, behavior, and practices related to the management of biomedical waste	Medical professionals, including doctors, nurses, and laboratory technicians, possess a more comprehensive understanding of biomedical waste management in comparison to sanitary staff. Specifically, nurses and laboratory personnel exhibit a greater proficiency in areas such as color coding and waste segregation at the point of origin, surpassing the expertise of doctors in these aspects.
4	Jarhyan et al. [15]	2012	PRHPs practicing in the villages of Comprehensive Rural Health Services Project (CRHSP)	Haryana	Profile and practices	80 private rural health providers (PRHPs) participated in the study. It has been observed that the majority of individuals practicing PRHPs do not possess any official credentials in the field of medicine.
5	Pant [16]	2012	Hospital staff	Dehradun	Practices in biomedical waste management	Small hospitals have poor waste management practices. Biomedical wastes are not properly segregated.
6	Singh et al. [17]	2012	Dentists in dental hospital	India	For the procedure of controlling cross-infection, knowledge, attitude, and practices	69% of people dispose of dangerous materials in public dumpsters, while 71% sterilize with hot water.
7	Joshi et al. [18]	2015	The studies in qualitative of rural tertiary care hospital	India	Perceptions of staff regarding the management of biomedical or healthcare waste	Challenges in integrating HCWM into organizational practice and interventions to enhance healthcare waste management (HCWM) were found to be the two main themes.
8	Ashwini and Hiremath [19]	2017	Private tertiary care hospital	Karnataka, India	Knowledge, attitude, and behaviors of paramedics and support workers	Knowledge regarding correct segregation of biomedical waste was 89.7% among nursing staff, 83.0% among ward support staff, and 25.6% among technicians, whereas it was correctly practiced by 87.1%, 57.4%, and 23.1% of nursing, ward support staff, and technicians, respectively.
9	Aanandaswamy et al. [20]	2019	Tertiary care center	India	Evaluation of knowledge, attitude, and practices regarding BMWM	Deficiencies in understanding and adherence to biomedical waste management protocols have been identified. There is a distinct necessity for comprehensive training programs designed specifically for personnel working within the operation room environment.
10	Rajan et al. [21]	2019	Ayurveda hospitals	India	Current practices and prospects	Categorization of biomedical wastes in Ayurvedic hospitals. Identification of future research areas in waste management.
11	Subramanian et al. [22]	2020	Dentistry	India	Practices in biomedical waste management (BMWM)	Most people are aware of the regulations and legislation governing the treatment of biological waste. Separating biomedical waste at the site of its origin is crucial.
12	Akkajit et al. [23]	2020	Healthcare workers in clinics	India	Knowledge, attitude, and practice with respect to medical waste management	Women made up the majority of the respondents (87.2%). The most common age range was 20–29 years old, accounting for 36.9% of the total population.
13	Kumar and Vaz [24]	2018	Tertiary care hospital	Goa, India	Biomedical waste management	96.5% of participants were aware of biomedical waste generation. 86.7% of participants practiced segregation of waste at the source.
14	Bhalla et al. [25]	2021	Tertiary care hospital	North, India	Knowledge, attitude, and practice regarding BMW management	The percentage of participants who provided satisfactory answers (60%) in the knowledge, attitudes, or practices domains showed a significant correlation (P = 0.001) between previous training and a more substantial percentage of correct responses, with faculty giving the best answers and support staff giving the worst.
15	Basavaraj et al. [26]	2021	Healthcare workers in a dedicated COVID hospital	Bangalore	Knowledge, attitude, and practices in biomedical waste management	The level of knowledge demonstrated by healthcare personnel can be deemed satisfactory. However, there is a noticeable scope for enhancement in the realm of practices pertaining to biomedical waste (BMW) management. This is particularly relevant among individuals classified within the Group D category of workers.
16	Sri et al. [27]	2023	A medical facility that prioritizes patient education and treatment amidst the COVID-19	India	Biomedical waste management	Almost 279 HCWs took part in the investigation and provided their opinions. Although different practices were observed among healthcare workers, the knowledge and attitude domain on the BMWM demonstrated statistical significance. Health professionals had an advantage over other healthcare workers who participated in procedures with varying attrition factors.

The management of biomedical waste is important for safeguarding both public health and environmental integrity, owing to the inherent hazards tied to the inadequate management of waste originating from healthcare establishments. In India, a rapidly growing healthcare industry coupled with increasing public health concerns has necessitated the implementation of effective BMWM practices [28].

Compliance and regulatory framework

Effective BMWM is promoted by a strong compliance framework and regulatory measures. This critically examines the role of compliance and the regulatory framework in shaping BMWM practices [29]. By delving into existing regulations, their enforcement, and potential challenges, this analysis illuminates the significance of regulatory adherence in safeguarding public health and the environment. Compared to smaller clinics and nursing homes, larger hospitals and well-established institutions generally showed better compliance [30]. To prevent health risks and environmental contamination, it is essential to ensure that all healthcare facilities strictly adhere to waste management regulations [31]. Compliance with BMWM regulations is necessary to ensure secure and efficient procedures [32]. The BMWM rules of 2016 were created under the Environment Protection Act and provide guidelines for managing, categorizing, processing, and disposing of biomedical waste [33].

Waste segregation and handling

Waste segregation and proper handling constitute the fundamental pillars of an effective BMWM system. Smaller facilities often lacked proper waste segregation procedures, while many larger hospitals had clearly defined segregation protocols [34]. Inadequate biomedical waste segregation puts waste handlers and healthcare workers at risk and makes the waste treatment process more difficult. To improve waste management procedures, training, and awareness programs must strongly emphasize proper waste segregation [35]. Waste generated within healthcare facilities encompasses a wide range of materials, each carrying varying degrees of risk. Proper waste segregation is essential to categorize waste into distinct streams, such as infectious, hazardous, and general waste [36]. Trained personnel handled hazardous waste, including sharp objects and infectious materials, using appropriate personal protective equipment (PPE). BMWM helped reduce the risk of occupational exposure and the spread of infections among waste-handling and healthcare workers [37].

Treatment and disposal methods

A crucial part of the management of biomedical waste is its treatment and disposal, which ensures that the waste produced in healthcare institutions is safe for the environment and human health. The Biomedical Waste Management Rules, 2016, which offer recommendations for secure and environmentally friendly ways to handle biomedical waste, regulate this process in India [38].

Treatment Methods

Incineration: Biomedical waste is burned under controlled conditions at high temperatures during incineration. In India, it is one of the most popular strategies. The waste is subjected to temperatures between 800°C and 1,200°C during incineration, effectively reducing microorganisms and reducing the volume of waste [39].

Autoclaving: A steam-based treatment approach is autoclaving. During this procedure, the waste is placed in an autoclave, which employs high-pressure steam to sterilize the waste and eliminate microorganisms [40].

Microwaving: Biomedical waste is heated and sterilized by microwave radiation. The efficiency of this relatively new technique in inactivating pathogens is attracting attention [41].

Chemical disinfection: Biomedical waste is disinfected using chemicals such as hydrogen peroxide or chlorine. It may not significantly reduce waste volume, even if it efficiently kills microorganisms [42].

Non-burn technologies: Environmentally friendly alternatives to incineration are emerging, including non-burn technologies like plasma gasification and encapsulation. These techniques reduce emissions while converting garbage into non-hazardous products or energy through heat or chemical processes [43].

Disposal Methods

Landfilling: After treatment, the residual material can be dumped in landfills designated for biomedical waste. The design of these landfills prevents toxins from leaching into the soil and groundwater. Proper lining and covering of waste is critical in landfill disposal [44].

Deep burial: In rare cases, waste that has been appropriately processed may be buried deeply in a secured pit. This technique is often reserved for waste that cannot be conveniently disposed of in another way [45].

Inertization: To contain any residual dangerous components, treated biomedical waste is mixed with inert substances such as cement or fly ash. This produces solid bricks or blocks that can be disposed of in landfills without any risk [46].

Lack of infrastructure in remote areas

In remote and underserved regions, such as rural areas in developing countries, healthcare facilities frequently face challenges due to inadequate infrastructure and resource shortages for effective waste management. To address these disparities, targeted interventions and investments in waste management infrastructure are needed in these regions, which have difficulty accessing waste treatment facilities, resulting in improper waste disposal and potential health risks [47]. The evaluation of healthcare facilities in India for our study revealed a significant challenge: the need for adequate infrastructure to manage biomedical waste in remote areas. Due to their isolation from other sites, lack of resources, and difficult access to waste management facilities, remote areas often experience unique challenges when managing biomedical waste. Targeted interventions are needed to effectively address the problem that arises from this circumstance [48].

Awareness and training

The emphasis on the necessity of continuing education suggests that the frequency of the present training programs may not be sufficient. Frequent training sessions are necessary to handle changing waste management concerns and reinforce existing knowledge. Expanding the training scope beyond specific procedures such as waste segregation, handling, and infection control to include emerging technologies and best practices could enhance its overall effectiveness. Addressing disparities in BMWM understanding among healthcare professionals requires tailoring training programs to accommodate varying levels of awareness and expertise. Highlighting the importance of environmental impact, training should integrate eco-friendly waste treatment methods to address the current gap in comprehensive waste management practices. Incorporating practical scenarios, simulations, or on-site training sessions can provide healthcare professionals with the skills and confidence needed to implement waste management practices effectively. Addressing these lacunae could contribute to a more robust and comprehensive training program for healthcare professionals involved in BMWM [49].

Collaboration and stakeholder engagement

BMWM involves multiple parties with crucial roles to ensure effective and sustainable waste procedures. We need to involve stakeholders and work together to establish effective BMWM systems [50]. Effectively addressing the difficulties and impediments in BMWM can be achieved through an integrated strategy involving all interested parties. Better waste management practices can be implemented by encouraging healthcare professionals' participation in decision-making processes [51].

Environmental impact

The management of biomedical waste raises significant environmental concerns. Ineffective waste management techniques and poor disposal procedures can contaminate soil and water, posing severe ecological risks. The analysis delves into the multifaceted challenges posed by improper BMWM. Inadequate waste segregation, untreated waste disposal, and outdated incineration methods can lead to air and soil pollution, contaminating ecosystems and water sources [52]. This underscores the need for comprehensive waste management strategies that mitigate environmental harm. Some medical facilities have made great attempts to reduce their impact on the environment. Compared to conventional incineration, these facilities have adopted environmentally friendly waste treatment techniques such as autoclaving and microwaving. These techniques allow these facilities to significantly lower the emissions of dangerous gases and particles [53].

## Conclusions

In the face of escalating biomedical waste challenges, there is no time for complacency. It is imperative that regulatory agencies, healthcare facilities, waste management authorities, and stakeholders unite to develop a comprehensive strategy to address India's BMWM issues. Through the implementation of evidence-based interventions and policy changes that are in line with international best practices, the study's insights are intended to assist healthcare leaders and legislators in improving the environment's safety and well-being. This approach may involve the development of comprehensive training programs to enhance the knowledge and skills of healthcare professionals in waste management practices. Additionally, strategic investments in infrastructure and technology can bolster waste treatment and disposal capabilities. Moreover, raising public awareness about the importance of proper BMWM through education campaigns and outreach initiatives can mobilize communities to actively participate in waste reduction and recycling efforts. By working together towards a shared vision of safe and sustainable waste management practices, we can safeguard the environment, protect public health, and secure a better future for generations to come.
